# Cytidine 5′-Diphosphocholine (Citicoline) in Glaucoma: Rationale of Its Use, Current Evidence and Future Perspectives

**DOI:** 10.3390/ijms161226099

**Published:** 2015-11-30

**Authors:** Gloria Roberti, Lucia Tanga, Manuele Michelessi, Luciano Quaranta, Vincenzo Parisi, Gianluca Manni, Francesco Oddone

**Affiliations:** 1 IRCCS-Fondazione GB Bietti, Via Livenza, 3, 00198 Rome, Italy; lucia.tanga@gmail.com (L.T.); vmparisi@gmail.com (V.P.); oddonef@gmail.com (F.O.); 2 DSMC, Università degli studi di Brescia, USVD “Centro per lo studio del Glaucoma” P.le Spedali Civili, 1, 25123 Brescia, Italy; luciano.quaranta@unibs.it; 3 DSCMT, Università di Roma Tor Vergata, Viale Oxford 81, 00133 Rome, Italy; gianlucamanni53@gmail.com

**Keywords:** citicoline, glaucoma, neuroprotection, retinal ganglion cells

## Abstract

Cytidine 5′-diphosphocholine or citicoline is an endogenous compound that acts in the biosynthetic pathway of phospholipids of cell membranes, particularly phosphatidylcholine, and it is able to increase neurotrasmitters levels in the central nervous system. Citicoline has shown positive effects in Parkinson’s disease and Alzheimer’s disease, as well as in amblyopia. Glaucoma is a neurodegenerative disease currently considered a disease involving ocular and visual brain structures. Neuroprotection has been proposed as a valid therapeutic option for those patients progressing despite a well-controlled intraocular pressure, the main risk factor for the progression of the disease. The aim of this review is to critically summarize the current evidence about the effect of citicoline in glaucoma.

## 1. Introduction

### Cytidine 5′-Diphosphocholine (Citicoline): Mechanism of Action

**Figure 1:**
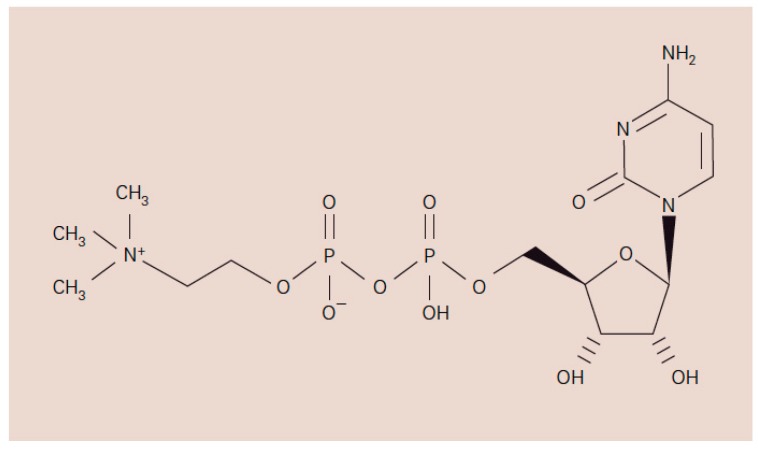
Chemical structure of Cytidine 5′-Diphosphocholine or citicoline [4].

Cytidine 5′-diphosphocholine, known as citicoline, is a naturally occurring endogenous compound. It is a mononucleotide composed of ribose, cytosine, pyrophosphate and choline (Figure 1) and it is an intermediate in the synthesis of membrane phospholipids such as phosphatidylcholine [1,2,3].

It has been reported that changes of citicoline concentration modify the synthesis of 80% of central nervous system (CNS) phospholipids.

Choline precursors are exogenous molecules that are converted in the body. Oral doses of citicoline are in fact absorbed rapidly and then hydrolysed in the intestinal wall and liver to choline and cytidine. Both choline and cytidine enter the systemic circulation, cross the blood–brain barrier, and combine to form citicoline within the CNS where it increases the biosynthesis of phospholipids [4]. Citicoline stimulates phosphatidylcholine synthesis, attenuates Free Fatty Acids (FFAs) release. Furthermore, citicoline re-establishes levels of cardiolipin phospholipid component of the inner mitochondrial membrane (structural action) [5].

Citicoline also increases some brain neurotransmitters such as dopamine (via enhancing tyrosine hydroxylase activity and inhibiting dopamine reuptake), noradrenaline, serotonin and serves as a choline donor in the biosynthesis of acetylcholine (functional action) [6].

Recently, citicoline has been studied in a murine model of experimental autoimmune encephalomyelitis and in the cuprizone model of toxic induced demyelination where promoted remyelination by increasing the number of oligodendrocyte precursor cell *in vivo* and *in vitro*, suggesting a new mechanism of action of this molecule with potential clinical impact in multiple sclerosis patients [7].

Citicoline can be considered a non-toxic molecule. In preclinical studies, none of the animals died after taking the maximum oral dose. Toxicity studies of oral citicoline did not show any toxic effects when it was administered in rodents and dogs whether for 30 days or for six months. Blood chemistry, organ histology, neurological or urinary parameters did not change [8].

In many clinical trials no serious adverse events have been provided, while after few days of treatment sporadic nonserious adverse effects included gastrointestinal discomfort, uneasiness, and irritability.

A large study analyzed the safety profile of citicoline in 2817 patients and only 5% of patients reported adverse effects related to the therapy and no patients needed to stop the therapy [9]. Those patients were affected with Alzheimer’s disease (AD) and vascular dementia and were treated for 2–9 weeks.

Because of all these properties, the use of citicoline has been studied as a promising therapeutic strategy in brain ischemia, AD and Parkinson’s disease (PD) and in ocular diseases such as amblyopia, non-arteritic ischemic optic neuropathy and glaucoma [10,11,12,13,14,15,16,17,18,19,20,21,22,23,24,25,26,27,28,29,30].

## 2. Evidence on the Effects of Citicoline in Ocular and Non-Ocular Neurological Conditions

### 2.1. Citicoline and Brain Ischemia

During brain ischemia and trauma there is an increase of activation of phospolipases A2 with accumulation of FFAs and arachidonic acid [12].

A large number of experimental studies have explored the protective effects of citicoline in stroke models. At the experimental level, citicoline has been reported to reduce infarct volume and brain edema. This led to an improvement of neurologic deficits either when citicoline was used as a single therapy or in combination with other agents [13].

Clinical stroke trials reported the effectiveness of citicoline when used soon after the ischemia, as demonstrated by improvements in level of consciousness and modified Rankin score (mRs). Nevertheless, since different sample sizes, multiple doses and several outcome endpoints were used, it became difficult to reach substantial conclusions.

In 2012 the results of an international, randomized, multicenter, placebo-controlled study (the ICTUS trial) initiated to confirm the efficacy of citicoline for the treatment of ischemic stroke in a larger trial, were published on the Lancet. In the study 2298 patients with moderate-to-severe acute ischemic stroke were randomly assigned in a 1:1 ratio to get citicoline or placebo within 24 h after the onset of symptoms (1000 mg every 12 h intravenously during the first three days and later orally 500 mg two times per day) for a total of six weeks. The primary outcome was recovery after 90 days measured by the results of three tests: National Institutes of Health Stroke Scale (NHSS), mRs and Barthel Index. The NHHS is a 15-item scale that measures the level of neurological impairment, the mRs is a measure of disability and Barthel index assesses the ability of patients to practice daily activities [14].

The results showed that global recovery was similar in both groups (odds ratio 1.03, 95% CI 0.86–1.25; *p* = 0.364) and the authors concluded that under the clinical conditions of the ICTUS trial, citicoline was not effective [14].

Recently, it has been highlighted that a large proportion of patients in the ICTUS trial has been treated with intra-venous recombinant tissue plasminogen activator (rt-PA) and this might have interfered with the potential beneficial effects of citicoline. Rt-PA in fact might have re-established blood flow in the penumbra region, and therefore, the benefit that citicoline could provide was altered demonstrating the difficulty of improving the outcome on top of the effect of rt-PA, caused by a ceiling effect of thrombolysis. The authors then concluded that the results of the ICTUS suggested that citicoline might be useful in those patients with acute ischemic stroke who were not treated with rt-PA [15].

### 2.2. Citicoline and Alzheimer’s Disease (AD)

Citicoline may hinder the deposition of beta-amyloid, a pivotal neurotoxic protein in the pathophysiology of AD [16].

It has been proposed an interaction between amyloid peptiteds formation and membrane phospholipid breakdown [17,18,19].

Amyloid peptides are able to activate phospholipase A2 [17]; therefore, formation of amyloid peptide is expected to accelerate membrane breakdown [18,19].

Alvarez *et al.* [20] have investigated the efficacy and safety of the treatment with citicoline *versus* placebo in 30 patients with AD after 12 weeks of treatment. It was a double-blind placebo-controlled study and compared to placebo, citicoline improved cognitive performance in AD patients, and this improvement was more pronounced in patients with mild dementia. Those patients showed also an increase in the percentage of brain bioelectrical activity and in cerebral blood flow velocity.

Franco *et al.* [21] confirmed an improvement in mental performance and brain electrical activity data correlated with cognitive parameters in patients with early-onset AD after one month of treatment with CDP-choline.

Furthermore, a recent Cochrane systematic review on the role of citicoline in cognitive impairment identified 14 trials enrolling 1051 patients with vascular cognitive impairment, vascular dementia or senile dementia (mild to moderate) and concluded that there was some evidence of a positive effect of the molecule upon memory, behavior, and global functioning, despite not on attention [22].

### 2.3. Citicoline and Parkinson’s Disease (PD)

Many authors have investigated the role of citicoline in PD [23,24,25] postulating that stimulation of the dopaminergic system induced by citicoline is at the basis of the improvement of neurological symptoms in PD.

Agnoli *et al.* [23] administered citicoline to PD patients in a double-blind cross-over controlled study. All patients were already treated with l-dopa + dopa decarboxylase inhibitor. Citicoline treatment determined a significant improvement of rigidity and bradykinesia and a less important improvement of tremor.

Besides, citicoline could be used to decrease the incidence of side effects and slow down the loss of efficacy of levodopa in the long-term treatment. In a study by Eberhardt R *et al.* [24], 85 patients with an established diagnosis of primary PD were randomly assigned to receive 381 mg of levodopa daily plus 1200 mg of citicoline daily or half dose of daily levodopa (mean, 196 mg daily) plus citicoline.

The results of the Webster Rating Scale, indicating the severity of the disease and the clinical impairment, and results of other tests evaluating the ability to walk, draw and write and a global clinical evaluation were performed before and after four weeks of treatment: no significant differences were found between the two study groups. Improvements of tests’ scores were shown in more patients who received half their levodopa dose plus citicoline than in those who continued to receive their usual levodopa dose plus the citicoline. Therefore, the authors concluded that citicoline could be used to decrease the incidence the side effects of levodopa and to delay the loss of its efficacy in the long-term treatment [25].

### 2.4. Citicoline in Amblyopia and Non-Arteritic Ischaemic Optic Neuropathy

The stimulation of the dopaminergic system by citicoline may be also responsible of the improvement of retinal and postretinal visual pathways in amblyopia.

Citicoline has been shown to enhance visual acuity, contrast sensitivity, visual evoked responses and the effect of part-time occlusion, at least temporarily, in amblyopic patients over the plastic period of the visual system.

Campos *et al.* [26] compared the effect of orally administered citicoline on visual function in addition to patching for the treatment of amblyopia in children after 30-day treatment. In an open-label parallel group study sixty-one participants suffering from anisometropic or strabismic amblyopia, citicoline was not found to be more effective than patching alone, but citicoline was able to stabilize the effect obtained during the treatment period. In fact, while after 90 days subjects treated only with patching showed a decrease in visual acuity, those receiving citicoline plus patching appeared to keep their visual acuity [27,28].

Porciatti *et al.* [29] measured visual acuity, contrast sensitivity and Visual Evoked Potential (VEP) in a group of 10 amblyopic subjects before 15 days of intramuscular treatment with citicoline, and the day after the end of treatment. On average, after treatment, visual acuity improved in both eyes, 1.4–1.5 lines in the amblyopic eyes and 0.4 lines in the control eyes. Contrast sensitivity improved in both dominant and amblyopic eyes of about 3 dB. VEPs increased in amplitude (about 30%) and advanced in phase (about 0.2 pi rad).

In 2008, Parisi *et al.* [30] evaluated visual function before and after treatment with oral citicoline for 60 days in patients with non-arteritic ischaemic optic neuropathy. At the end of treatment, patients showed an improvement (*p* < 0.01) of Pattern Electroretinogram (PERG), VEP parameters and visual acuity, compared to pre-treatment values. After wash out, functional improvements persisted compared to baseline. The age-matched control group did not show any improvement.

## 3. Glaucoma as a Central Nervous System (CNS) Disease

Glaucoma is a group of optic neuropathies in which the death of retinal ganglion cells (RGCs) leads to an abnormal increase of the optic nerve head (ONH) excavation and a correspondent permanent impairment of visual field [31]. It represents the second cause of irreversible blindness in the world and 111.8 million of people are estimated to be affected in 2040 [32]. An intraocular pressure (IOP) elevated beyond the susceptibility of retinal structure, is considered the most important risk factor for developing glaucoma, and several randomized clinical trials have shown that untreated patients with elevated IOP are more likely to develop glaucomatous damages than those receiving treatment [33,34,35].

Despite lowering IOP by medical therapy, laser or surgical procedures is the only approach generally recognized, up-to-date there is a body of evidence that suggests IOP is not the unique risk factor involved in the pathogenesis of the disease.

Elevated IOP is not always associated with glaucoma considering that a certain amount of subjects with an IOP beyond normal statistical limits will never develop the disease [36]. On the other hand, hypotensive therapy alone is not always sufficient in preserving visual function, considering that in some cases, despite a well-controlled IOP the disease continue to progress [37].

We can assume that in such patients, a more consistent IOP lowering would be beneficial and needed, but it is also reasonable to hypothesize that other factors rather than IOP are involved.

Glaucoma was originally identified as a disease associated with IOP and for long-time elevated IOP was considered synonymous to glaucoma. To date, IOP is still managed as a the main risk factor but current glaucoma definition is focused on physiopathological features of the disease [37,38]: a progressive optic neuropathy, which primarily involves RGCs, and concurrently alters the whole visual pathways running from the eye to visual cortex.

Gupta *et al.* [39] in a 2006 study have firstly demonstrated the presence of degenerative changes at lateral geniculate nucleus (LGN) and visual cortex in a human glaucoma case. RGCs death was associated with atrophy and loss of their target neurons at the level of LGN and visual cortex thickness was reduced compared with controls. These results were in accordance with previous histological evidence from experimental animal model studies [40,41].

Therefore, the eye could be viewed as a part of the CNS and correspondingly, glaucoma could be considered a brain neurodegenerative disease, as firstly suggested by Shumer *et al.* [42] in a 1994 study. Moreover, neurological progressive diseases, such as AD, amyotrophic lateral sclerosis and PD, exhibit several common mechanisms of cell death with glaucoma.

The paradigm of glaucoma like as a neurological disorder, associated with evidences showing how insufficient is the therapeutic approach based on the IOP lowering therapy alone, suggests that neuroprotection may play a therapeutic role (alternative or adjuvant) in glaucoma.

## 4. Glaucoma Physiopathology and Neuroprotection

Even though pathological changes in glaucoma occur along the whole visual pathway, the death of the RGCs is the crucial pathophysiological event [43,44].

Moreover, the damage of RGCs is not limited to the neurons primary insulted, as a matter of fact a secondary degeneration may also involve the neighboring neurons localized closely to those initially damaged, as a consequence of extracellular environment changes. Degeneration also occurs in the target neurons by a process called trans-synaptic degeneration. Such forms of slow and progressive secondary or trans-synaptic degeneration, may explain why lowering IOP is not always enough to halt the progression of the disease in glaucoma patients.

Whatever the primary insult is, the final death pathway of RGC is a programmed cell death process called apoptosis [45,46].

Neuroprotection is a therapeutic approach aimed to prevent, hamper, delay or reduce neuronal cell death by directly targeting neurons [47]. Differently from IOP lowering strategy, which prevents RGCs death by reducing primary insult (indirect neuroprotection), neuroprotective agents aim to influence the biological properties of the neurons that are directly involved in cell death pathway. Such form of “cell protection” could be effective whatever the primary insult is located, and even when the primary insult itself is not completely or partially known, because it is directed toward the final common mechanism involved in RGCs death.

RGCs survival is believed to be dependent from a balance between survival signals and neurotoxic stimuli. Apoptosis is the consequence of the lost of such a balance, which is shifted in favor of neurotoxic stimuli. Neuroprotective agents can act either by increasing the survival stimuli and/or by hindering the neurotoxic signal, and more in general by targeting the mechanisms that trigger the apoptotic cascade [48].

Deprivation of neurotrophins is one of the mechanisms involved [49]. In glaucoma acute or chronic elevation of IOP can lead to a blockade of the axonal retrograde transport of neurotrophin from the superior colliculus to the ONH [50,51]. Neurotrophic factor (such as brain derived neurotrophic factor, BDNF) are essential for cell survival and growth and apoptosis might be a consequence of their deprivation. A neuroprotective approach relying on the increase of endogenous expression or providing exogenous intake of neurotrophins, has been assessed in several studies [52,53].

Another mechanism largely investigated is the glutamate-mediated excitotoxicity. Cells that start undergoing apoptosis usually release a large amount of glutamate in the extracellular area, which increases the income of Ca^2+^ in the neighboring cells, leading to secondary degeneration [54]. Blocking the glutamate excitotoxicity cascade, by interfering with its release or blocking its receptor, represents a potential neuroprotective strategy [55].

Protein misfolding, oxidative stress, mithocondrial dysfunction, activation of astrocytes and microglia are further mechanisms suggested to be involved as triggers of the apoptotic cascade and potential targets for neuroprotection. Despite none of them have achieved a predominant role, a large amount of pre-clinical studies have shown promising results on the effectiveness of several neuroprotectant molecules in reducing the loss of RGCs in glaucoma experimental models [56,57].

For example, recent studies have proposed methylene blue as a neuroprotectant. This agent might interfere with the mitochondrial electron transport complex reducing production of superoxide and free radicals in response to the stress induced by glutamate [58]. In addition, the 17β-estradiol (E2) has been investigated to prevent RGC death in a glaucoma model of elevated IOP in rats. After treatment with E2 eye drops the number of apoptotic cells in the RGC layers was significantly decreased but systemic side effects could not be prevented [59].

Unfortunately, translation from pre-clinical studies to randomized clinical trials is still lacking, and up-to-now only two neuroprotective drugs are currently approved by the Food and Drugs Administration: memantine for AD (moderate to advance stages) [60] and riluzole for lateral amyotrophic disease [61]. For glaucoma, instead, no neuroprotectans have been registered so far.

## 5. Role of Citicoline in Glaucoma: Current Evidence

### 5.1. Experimental Studies on Citicoline as a Neuroprotectant

Some experimental studies have confirmed the protective role of citicoline on RGCs and its neuromodulator effect (Table 1).

In 2002 Rejadak *et al.* [62] showed that citicoline influences retinal catecholamine levels in adult male Albino rabbits. Six animals were injected intraperitoneally with 50 mg/kg of citicoline twice daily for seven days. The control group (four animals) received vehicle injections according to the same schedule. At the end of the treatment period animals were sacrificed and retinal catecholamine concentrations were measured using HPLC. Dopamine levels were found to be higher in animal treated with citicoline compared to those treated with vehicle only, while adrenaline concentration was slightly higher and noradrenalin remained unmodified.

Oshitari *et al.* [63] aimed to study the clear effect of citicoline on damaged RGCs. They carried out tissue culture of mouse retinal explants, and investigated the effect of citicoline on damaged RGCs by quantitative analysis of TdT-dUTP terminal nick-end labeling (TUNEL) positivity in the ganglion cell layer (GCL) and the assessment of the amount of regenerating neurites. The proportion of TUNEL-positive cells in the GCL was very low in mouse retina treated with 0.1–10 mmol/L compared with those of the control (*p* < 0.05).

Authors postulated an antiapoptotic effect of citicoline in mitochondria-dependent cell death mechanism, and its ability in supporting axon regeneration.

The antiapoptotic effect of citicoline was also evaluated in the study by Shuettauf *et al.* [64]. Adult rats were divided in four groups and treated with vehicle only, with citicoline (1 g/kg the first seven days and then 300 mg/kg), with lithium (30 mg/kg) or with a mixture of citicoline and lithium. Intraperitoneal injections of drugs were made once a day, starting one day prior to partial optic nerve crush, up to the day of the tissue sampling. The density of RGC connected with superior colliculus was higher in animals treated with citicoline compared to those treated with vehicle one week and three weeks after the crush. The expression of the antiapoptotic protein Bcl-2 was recorded in the lithium group, in the citicoline group and in the group treated with both drugs [64].

**Table 1:** Experimental studies.

Authors	Year	Study Design	Animal	Citicoline Concentration	Citicoline Administration	Outcomes
Rejdak R *et al.* [62]	2002	case-control	Albino rabbits	50 mg/kg/twice day	Intraperitoneal injection	Retinal catecholamine levels
Park CH *et al.* [65]	2005	case-control	Spraue-Dawley rats	50 mg·kg^−1^	Intraperitoneal injection	Thickness of retinal layers and expression of ChAT and TH
Schuettauf F *et al.* [64]	2006	case-control	Rats	1 g/kg/daily and 300 mg/kg/daily	Intraperitomeal injection	Retinal ganglion cells density and expression of the antiapoptotic protein Bcl-2
Oshitari T *et al.* [63].	2010	case-control	Cultures from Spraue-Dawley rats	1 μM	Added to high glucose medium in retinal culture	Apoptosis evaluation by TUNEL assay and Caspase-3 and Caspase-9 activity
Matteucci A *et al.* [66].	2014	case-control	Cultures from embryonic rat retina	10, 100 and 1000 μM	Treated for 96 h and 24 h before glutamate-induced excitotoxic insult and high glucose-promoted neuronal cell damage	Apoptosis evaluation by TUNEL assay and caspase activation

Summary of experimental studies evaluating the effect of citicoline on retinal ganglion cells survival.

The neuromodulator effect of citicoline was observed in a model of Kainic acid (KA)-induced retinal damage, using choline acetyltransferase (ChAT) and tyrosine hydroxylase (TH) [65]. One group of male rats were injected with KA in the vitreous space, one group was injected intraperitoneally with citicoline (500 mg·kg^−1^) twice daily for one, three and seven days after KA-injection and one group served as control group. In KA treated animals the entire retinal thickness gradually decreased compared with the control retinas, while in the citicoline treated animals there was a significant attenuated reduction. Furthermore, the ChAT immunoreactivity was significantly protected by the treatment with citicoline, which prevented the complete disappearance of TH immunoreactivities. Since KA is an analogue of glutamate, authors concluded that citicoline might have the neuroprotective action in glutamate-mediated cell death [65].

In addition, the safety characteristic of the drug has been confirmed in *in vitro* models of retinal neurodegeneration.

In 2014, Matteucci *et al.* [66] investigated the effect of citicoline (10, 100 and 1000 μM) on cell damage in cultures from embryonic rat retina composed of mixed neuronal and glial cell population. Results confirmed that citicoline does not modify the rate of apoptotic cells, evaluated by TUNEL positivity and caspase activation and does not change cell composition. Then, authors set up models of both glutamate-induced excitotoxicity, considered of pathophysiological relevance in glaucoma, and high glucose-promoted neuronal cell damage. Primary retinal cultures were treated with 100 μM citicoline 24 h before excitotoxic insult, induced with exposure to 100 μM glutamate for 25 min. Controls included retinal cultures treated with iso-osmolar mannitol. The apoptotic rate evaluated by TUNEL assay and synaptic damage in glutamate-treated cells was significantly reduced by treatment with citicoline, compared to controls. The cell culture model used in this study allowed the evaluation of the effects of citicoline on different cell types and strengthened the neuroprotective effect of the intact citicoline. In fact, in *in vitro* system the drug was not metabolized and this evidenced why liposomal preparations of citicoline, where the drug is delivered as intact molecule, are more active than parenteral administration, which are more susceptible to transformation in the two metabolites choline and cytidine.

Recently, the neuroprotective effect of citicoline has been demonstrated on retinal nerve fibers in hyperglycemia conditions. In a mouse model of diabetes it has been shown that citicoline eye drops counteracts retinal nerve fibers layer thinning [67]. Results are in agreement with those of a previous study investigating the effect of BDNF, neurotrophin-4 (NT-4), and citicoline on neuronal apoptosis and neurite regeneration in cultured rat retinas exposed to high glucose. The authors, in fact, observed a decreased number of apoptotic cells with suppression of caspase-9 and caspase-3 activities [68].

### 5.2. Clinical Studies

The neurotrophic effect of citicoline in glaucoma management has been clinically investigated from long time (Table 2).

Pecori Giraldi *et al.* [69] in 1989 reported positive results obtained on visual fields of patients affected with open angle glaucoma after intramuscular injection of citicoline. The drug was given at the dose of 1 g for 10 consecutive days, and patients were examined at baseline and after 15 days and three months. The authors found an improvement of visual field in 75% of the 34 examined eyes, based on the reduction in the scotomatous area (computerized central perimetry) and mean defect. Visual field improvement remained stable for further three months with lack of side-effects and the same favorable results were obtained when the treatment was repeated.

Ten years later, the same authors reported the results from a prospective study on 23 eyes of 23 glaucoma patients with early or moderate perimetric defects with 10 year follow up [70]. Eleven patients were treated with citicoline administered intramuscularly associated with topical hypotensive treatment (1 g for 15 days repeated every 6 months), while 12 patients received topical hypotensive treatment alone. The outcome measure was visual field progression evaluated by an increase of the non-perception area (NPA) >500 mm^2^. Citicoline-treated patients exhibited a significant improvement of retinal sensitivity (NPA reduction) at one-year follow up, and remained stable after nine years.

The authors related these results to those obtained from neurological studies on the use of citicoline in ischemic, traumatic or degenerative events of the CNS and its hemodynamic properties, able to increase the cerebral and ocular blood flows.

To better understand which structures of the visual system may selectively participate in the improvement of damaged visual field, Parisi *et al.* [71], in 1999, evaluated the effect of citicoline on retinal function and on cortical responses in patients with glaucoma using electrofunctional tests (VEP and PERG). Forty patients with early to moderate glaucoma and well-controlled IOP were randomly divided into two age-matched groups: 25 were treated with citicoline plus hypotensive treatment (GC) and 15 were treated with placebo (GP) plus hypotensive treatment. A daily intramuscular dose of 1 g citicoline or placebo was prescribed for 60 days followed by 120 days of washout. Later, GC patients were divided in two age-matched groups: in 10 patients (GC1) the washout was prolonged for a further 120 days, while in 15 patients (GC2) a second 60-day period of citicoline treatment was followed by a second 120-day period of washout. The GP group showed similar parameters in all tests performed. In GC group, treatment with citicoline induced a significant improvement of VEP and PERG parameters that was *treatment-dependent*: at day 300 GC1 patients presented an additional worsening in VEP and PERG parameters and no differences with respect to baseline and with respect to GP patients, while in GC2 a second 60 days period of citicoline treatment induced an additional improvement of VEP and PERG parameters still maintained after the second washout.

In 2005, Parisi [72] reported data obtained from 12 GP and 12 GC patients during 14 additional periods of the schedule treatment (two months treatment with citicoline plus hypotensive therapy, four months washout from citicoline) for a total of 96 months (eight years). They wanted to evaluate the long-term effect of citicoline treatment, assessing the differences observed at the end of each period of follow up in comparison with baseline conditions. The additional periods of citicoline treatment in GC patients during the subsequent months induced a greater improvement of VEP and PERG parameters as compared with pre-treatment conditions, and when compared to GP patients. Furthermore, at the end of the follow up an increase in visual field mean deviation (MD) when compared to baseline values was also observed in all GC patients. This increase was significantly related to electrofunctional results.

They concluded that previously reported changes of perimetric conditions might be tributable to an improvement of both retinal and post-retinal structures induced by citicoline treatment. Since citicoline is able to increase dopamine levels in CNS. The authors suggested that in both VEP and PERG improvement a dopaminergic-like activity could be suggested. Dopamine is one of the most important neurotrasmitter involved in retinal and post retinal visual pathways and levodopa was found to increase retinal functions in humans treated with this substance [23,24,25]. Similar results were observed in amblyopic patients by the improvement of visual acuity after treatment with citicoline [26,27,28,29].

All these studies refer to citicoline treatment administered by intramuscular injection. Oral administration of citicoline was thereafter also investigated.

In particular, the first results were obtained from Rejadak *et al.* [73] in 2003. Eleven glaucoma patients were treated with citicoline tablets, each containing 0.5 g of the active ingredient. These tablets were prepared by the local hospital pharmacy. After the first VEP recording, the patients were supplied with 28 citicoline tablets (one tablet every 12 h for 14 days). After a two-week break, they were supplied with a second set of 28 citicoline tablets. The second VEP measurement was taken two weeks after the end of the second two-week treatment course. There was a significant positive correlation between the pretreatment VEP P100 latency values and the decrease of latency observed following treatment, and a significant difference in the pre-treatment VEP latencies between the responding and non-responding eyes. The authors concluded that oral citicoline treatment might normalize VEPs in glaucoma patients. In 2008 Parisi *et al.* [74] studied the effect of oral suspension (1600 mg/die) or intramuscular (1 g/die) citicoline on visual pathways function and conduction of glaucoma patients with moderate visual defects. Three groups of 20 patients under hypotensive therapy were followed for one year: Group 1 did not take citicoline, Group 2 took intramuscular citicoline and Group 3 took oral citicoline. The pharmacological treatment was performed for two months followed by four months of washout and the scheme was repeated for two times. Improvement of retinal function evaluated by PERG and neural conduction along visual pathways evaluated by VEP were observed after both oral and intramuscular treatment with citicoline. No differences were found between intramuscular and oral administration, and adverse side effects were not reported by any of the patients enrolled in the study. Partial regression was detected after washout.

**Figure 2:**
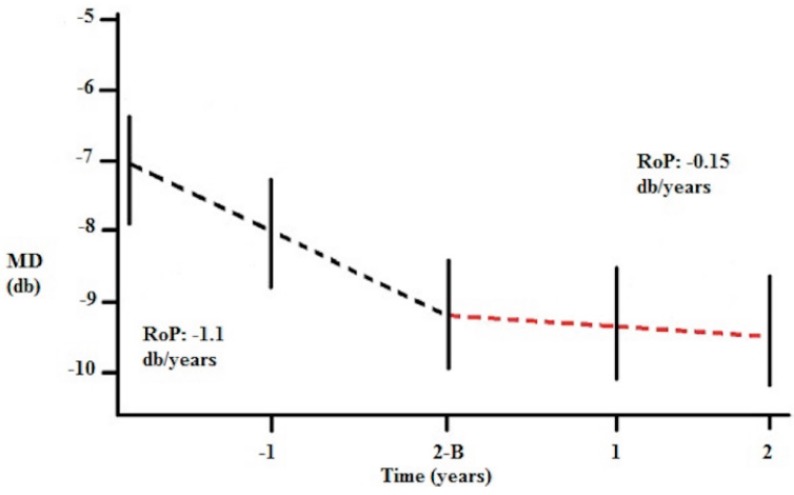
Rates of progression (RoP) during the study. B = Baseline. 2-B: two years before baseline. (Figure adapted from Reference n [75]).

In 2013, Ottobelli *et al.* [75] reported results from a multicentric study on the effect of citicoline oral solution on the rate of progression of visual field in patients with progressive glaucoma. Forty-one patients with a disease progression of at least −1 dB/year at MD for at least three years before entering the study despite controlled IOP completed the study. They received citicoline oral solution for two years (one vial, 500 mg per day for a period of four months and stopped for two months) and were followed up with four visual field tests per year for two years. From the first cycle of treatment with citicoline, the mean rate of progression significantly reduced to −0.15 (±0.3) dB/year at the end of the study (Figure 2).

Furthermore, IOP was approximately 1 mmHg lower during the follow up as compared to baseline, and patients with IOP higher than 15 mmHg had faster rate of progression (−0.25 dB/year) than patients with IOP lower than 15 mmHg (−0.05 dB/year). Citicoline and mean IOP were the only variables that were significantly associated with MD changes. This study confirmed the lack of side effects associated with citicoline even administered as oral solution.

Moreover, previous studies demonstrated that the oral solution has a good bioavailability and can reach the same therapeutic efficacy with respect to the parenteral administration (intravenous and intramuscular) [76,77].

**Table 2:** Clinical studies.

Authors	Year	Study Design	Study Population	Adm	Dosage	Schedule of Treatment	Follow-up	Outcomes
Pecori Giraldi *et al.* [69]	1989	cohort	Open Angle Glaucoma (OAG)	IM	1 g/day	10 days	3 months	Reduction in the scotomatous area (computerized central perimetry) and decrease in mean defect (automated perimetry)
Parisi V *et al.* [71]	1999	case-control	OAG −3 dB > Mean Deviation (MD) < −6 dB	IM	1 g/day	60 days 120 days of washout (2 cycles)	360 days	Visual Evoked Potential (VEP) and Pattern Electroretinogram (PERG) parameters
Virno M *et al.* [70]	2000	case-control	OAG	IM	1 g/day	15 days 180 days of washout (20 cycles)	10 years	Visual field worsening (increase of non-perception area >500 mm^2^)
Rejdak *et al.* [73]	2003	cohort	OAG	Oral	1 g/day	14 days 2 days of washout (2 cycles)	56 days	VEP parameters
Parisi V [72]	2005	case-control	OAG −3 dB > MD < −6 dB	IM	1 g/day	60 days 120 days of washout (14 cycles)	8 years	VEP and PERG parameters
Parisi V *et al.* [74]	2008	case-control	OAG −2 dB > MD < −14 dB	IM Oral	1 g/day 1600 mg/day	60 days 120 days of washout (2 cycles)	360 days	VEP and PERG parameters
Ottobelli L *et al.* [75]	2013	retrospective cohort	progressing OAG	Oral (solution)	500 mg/day	120 days 60 days of washout (4 cycles)	2 years	Rate of visual field progression
Roberti *et al.* [78]	2014	experimental and clinical (case-control)	OAG −3 dB > MD < −12 dB	Topic (eye drops)	3 drops/day	60 days	90 days	VEP and PERG parameters
Parisi V *et al.* [79]	2015	case-control	OAG MD > −10 dB	Topic (eye drops)	3 drops/day	120 days 60 days of washout	180 days	VEP and PERG parameters

Summary of clinical studies evaluating the effect of citicoline in glaucoma patients by means of visual field and electrophysiological parameters. Adm = administration. IM = intramuscular.

### 5.3. Citicoline Eye Drops in Glaucoma

To promote patient adherence and compliance to neuroprotective treatment, citicoline was also recently made available as eye solution. The results from an experimental study demonstrated that the molecule is traceable in the vitreous when administered topically in solution with benzalkonium chloride and hyaluronic acid [78].

In this study the right eyes of five CD1 mice were treated with two drops per day for three days of citicoline 1% and 2%. At the end of the treatment, the vitreous was analyzed using liquid chromatography and spectrometry mass (LC-MS/MS). Citicoline was detected in the vitreous and systemic absorption was also noted when the higher concentration was used (2%).

The second phase of this study was clinical, in order to determine if topical citicoline is able to delay glaucoma progression, considering visual field indices and electrofunctional tests. Patients were randomized in two groups, the first (16 patients) treated with citicoline eye drops plus ocular hypotensive therapy for two months and one month of washout and the second (18 patients) treated only with ocular hypotensive treatment for three months. Individual eyes of patients treated with citicoline eye drops showed positive improvement that was not statistically significant in the entire group. RGC function improved up to 30 days after the washout as shown by PERG parameters while VEP and retino-cortical time improvement regressed after 30 days of washout. No minor or serious adverse events were reported. No significant modifications of the IOP were recorded during the study. Authors concluded that citicoline eye drops seems to have a neuroprotective effect that is independent of pressure-lowering effect due to any hypotensive drops.

Recently, Parisi *et al.* [79] reported additional results of a prospective, randomized study on the retinal function and the neural conduction after treatment with citicoline eye drops in patients with open-angle glaucoma. One group of patients was treated with topical citicoline 3 drops/day in addition to the topical hypotensive treatment with beta-blockers for four months followed by a two-month washout period. The other group of patients was treated only with beta-blocker monotherapy for the whole study (six months). The electrophysiological examination was performed at the baseline and after four and six month of follow-up. The results of the present study confirmed that after treatment with citicoline eye drops a significant improvement in PERG and VEP parameters was recorded. In particular, the shortening of VEP P100 implicit times was correlated significantly with the increase of PERG P50-N95 amplitudes. Furthermore, a significant correlation was found between the values of the PERG amplitude at the baseline and relative differences suggesting that patients who had greater benefits from citicoline eye drops treatment were those that displayed the worst retinal function at baseline. After washout PERG and VEP values were similar to baseline levels, while patients treated exclusively with beta-blocker did not show significant changes of PERG and VEP values during the whole follow-up. Moreover, the improvement of the electrophysiological indices was found to correlate with the improvement of visual field mean deviation (patients treated with citicoline eye drops in addition to beta-blockers showed a positive mean progression rate of +0.56 dB while patients treated only with beta-blockers had a mean progression rate of −0.24 dB). None of the patients enrolled in the study reported any ocular side effect or changes in clinical parameters (IOP and visual acuity).

## 6. Comments and Future Perspectives

Despite the lack of studies on experimental optic nerve damage obtained by means of acute elevation of IOP, it has been validated that citicoline is able to protect RGC from apoptosis in glutamate-induced excitotoxicity animal models and after partial optic nerve crush [63,64,65,66]. Furthermore, wishing to test a neuroprotective agent among patients who are already maximally treated with hypotensive drugs, it might be less effective to test them in an IOP generated model.

In glaucoma management, the neuroprotection staircase has three main steps: to *protect* still undamaged axons and ganglion cells, to rescue minimally damaged axons and ganglion cells and to *regenerate* damaged axons and ganglion cells. In this scenario it may be hypothesized that citicoline may act in the second step. In particular, it might act in the window between dysfunction and death of RGC [80]. Animal models clearly demonstrate that RGC death occurs only at the late stages of the disease: axon transport slows first, axon severing is observed as second, and RGC death occurs later [81]. Thus the window between dysfunction and death provides a space for therapies inducing an increase of retinal elements functioning (neuroenhancement) to take effect and citicoline may play this role in the short-term period of treatment.

RGC dysfunction *versus* death cannot be distinguished by current visual field testing or optic nerve structural measurement, while PERG is able to demonstrate reversible dysfunction after acute pressure lowering in glaucoma patients [82].

The scientific literature on the role of citicoline in glaucoma management is growing and published studies differ in terms of populations’ characteristics (e.g., spectrum of visual field defect), outcome measures (most focused on electrophysiological parameters), length, administration, dosage and schedule of treatment. Despite of these differences, the available studies are consistent in indicating that the use of citicoline is associated with positive effects on the visual function. Furthermore, all the studies mentioned have not reported any adverse effects among patients enrolled confirming that this molecule is safe and can be used for long-term treatment.

## 7. Conclusions

The ideal neuroprotection study design is not easy to be realized due to the natural history of glaucoma itself [83]. This disease has a long, slow course with variable worsening rates among patients and high outcome measurement variability.

In the future, randomized clinical trials with large population and outcomes related to the patients’ quality of life (such as differential retinal sensitivity as measured by standard achromatic perimetry) are also needed to obtain a dose-response relation, and to increase the chances of therapeutic success. The morphological parameters by means of optical coherence tomography (*i.e.*, retinal nerve fiber layer, or ganglion cell complex thickness) should also be investigated to detect the clinical effect of citicoline.
